# Non-invasive real-time autonomic function characterization during surgery via continuous Poincaré quantification of heart rate variability

**DOI:** 10.1007/s10877-018-0206-4

**Published:** 2018-10-03

**Authors:** Maddalena Ardissino, Nicoletta Nicolaou, Marcela Vizcaychipi

**Affiliations:** 1 Imperial College School of Medicine, Imperial College London, London, SW7 2AZ UK; 2 University of Nicosia Medical School, 21 Ilia Papakyriakou, Egkomi, 2414 Nicosia, Cyprus; 3 Department of Electrical and Electronic Engineering, Imperial College London, London, SW7 2AZ UK; 4 Biomedical Engineering, School of Biological Sciences, University of Reading, Reading, RG6 6AY UK; 5 Magill Department of Anaesthesia, Intensive Care and Pain Management, Chelsea and Westminster Hospital, 369 Fulham Road, London, SW10 9NH UK

**Keywords:** Intraoperative monitoring, Real-time monitoring, Autonomic function, Poincaré, Heart rate variability

## Abstract

Heart rate variability (HRV) provides an excellent proxy for monitoring of autonomic function, but the clinical utility of such characterization has not been investigated. In a clinical setting, the baseline autonomic function can reflect ability to adapt to stressors such as anesthesia. No monitoring tool has yet been developed that is able to track changes in HRV in real time. This study is a proof-of-concept for a non-invasive, real-time monitoring model for autonomic function via continuous Poincaré quantification of HRV dynamics. Anonymized heart rate data of 18 healthy individuals (18–45 years) undergoing minor procedures and 18 healthy controls (21–35 years) were analyzed. Patients underwent propofol and fentanyl anesthesia, and controls were at rest. Continuous heart rate monitoring was carried out from before aesthetic induction to the end of the surgical procedure. HRV components (sympathetic and parasympathetic) were extracted and analyzed using Poincaré quantification, and a real-time assessment tool was developed. In the patient group, a significant decrease in the sympathetic and parasympathetic components of HRV was observed following anesthesia (SD1: p = 0.019; SD2: p = 0.00027). No corresponding change in HRV was observed in controls. HRV parameters were modelled into a real-time graph. Using the monitoring technique developed, autonomic changes could be successfully visualized in real-time. This could provide the basis for a novel, fast and non-invasive method of autonomic assessment that can be delivered at the point of care.

**Electronic supplementary material:**

The online version of this article (10.1007/s10877-018-0206-4) contains supplementary material, which is available to authorized users.

## Introduction

**Fig. 1:**
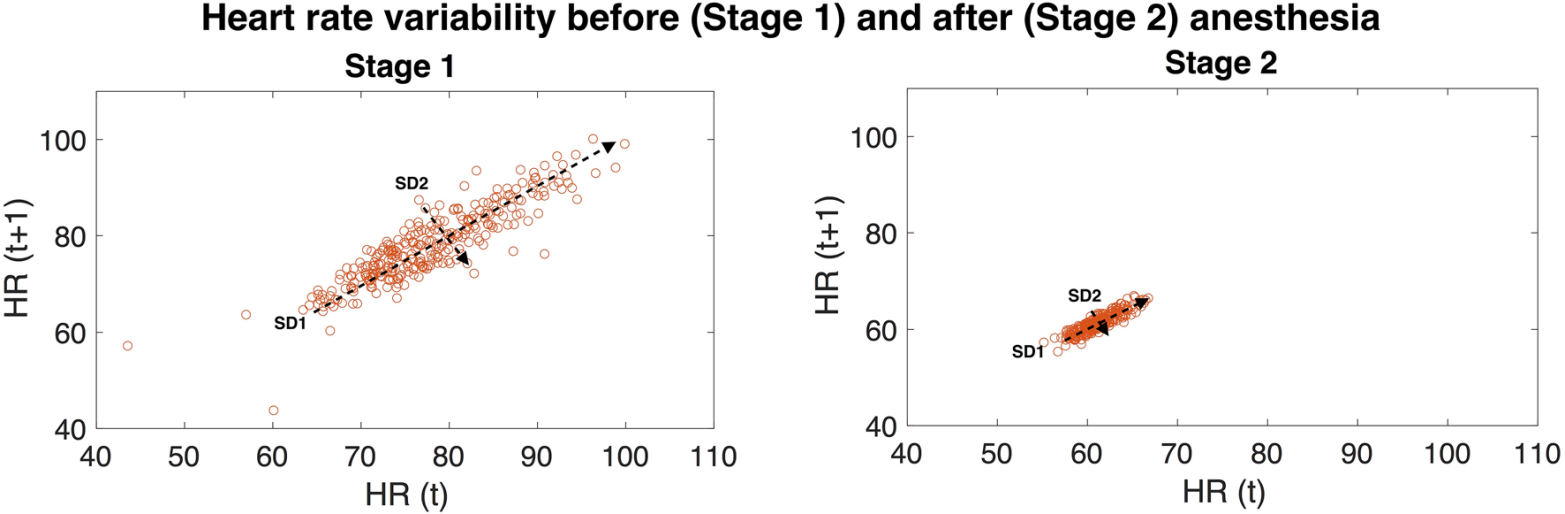
Poincare plots before (‘Stage 1’—left) and after (‘Stage 2’—right) anesthesia. Stage 1 anesthesia illustrates baseline data and it is defined as 5 min of recording prior to induction of anesthesia. Stage 2 anesthesia illustrates maintenance of anesthesia and it consists of 5 min taken during anesthesia. Changes in the plot shapes indicate changes in parasympathetic (SD2) and sympathetic (SD1) tone. HR(t) indicates the HR at the first beat, whereas HR(t + 1) indicates the HR of the next heartbeat; each data point therefore represents the relationship between HR of two successive heart beats

The autonomic nervous system (ANS) consists of two main components, the sympathetic and the parasympathetic nervous system (SNS and PSNS respectively), which are responsible for a wide variety of multisystem homeostatic changes, and play a part in the modulation of heart rate variability (HRV). Heart rate variability (HRV) provides an potential proxy for characterization of autonomic nervous system function. Ultimately, variability in heart rate results from continuous modulation of the sino-atrial node (SAN) by the autonomic nervous system, which varies in response to multiple factors such as respiratory rate [1], homeostatic reflexes and centrally generated physiological patterns. Together, these factors influence the sympathovagal balance; it is this balance that ultimately defines the heart rate variability [2]. In a clinical setting, impairments in autonomic function [3, 4] may be reflected by changes in heart rate variability [2]. As autonomic dysregulation is a major risk factor for complications of anesthesia such as bradycardia and hypotension [5–8], information regarding the autonomic function of patients both before and during anesthesia can be of great value to anesthetists. Thus far, no routine, point-of-care monitoring system has been developed to assess autonomic function in patients before and during anesthesia. Heart rate variability has been shown to mirror changes in autonomic function [9, 10], which can be evaluated through the use of Poincaré plots. Poincaré plots are non-linear, geometrical representations of HRV dynamics over a period of time [11, 12], in which the HR value at a given time, HR(t), is plotted against the value of the next HR value, HR(t + 1) throughout the duration of the recording (Fig. 1) [12]. Each point therefore represents the relationship between two consecutive heartbeats, thus providing a visual representation of beat-to-beat variability over time. Despite the validation of this technique as means of analyzing HRV [13, 14], it remains to be applied clinically.

This study sets out to provide an initial proof-of-concept for a novel tool that utilizes HRV as a surrogate measure of autonomic function to provide real-time, accurate and non-invasive measurement of autonomic function that can be delivered at the point of care.

## Method

### Ethical approval

Ethical approval for data collection in this study was granted by the West Midlands Research Ethics Committee (NHS REC ID: 14/WM/0179, IRAS project ID: 156151). It was also conducted according to the UK Good Clinical Practice in Research (Research Governance Framework for Health and Social Care 2005) and Patients Protection Act 1998.

### Patients

Anonymized continuous heart rate data recordings of 18 young and healthy (18–45 years) patients undergoing propofol anesthesia for arthroscopic surgery were used for analysis. Anesthesia was induced using a standardized protocol of propofol (3.5 mg/kg ± 1.3) followed by fentanyl (1.6 mcg/kg ± 0.7), and a record was made of any further interventions, such as fluid challenges and vasoactive drug administration. All of the participants received positive pressure ventilation. The depth of anesthesia in patients was monitored using a BIS™ monitoring system with a target of 40–60.

Control data was extracted from the freely available online Fantasia dataset [15] from the Massachusetts institute of Technology (MIT), that includes anonymized heart rate data from healthy young volunteers (21–35 years) at rest. Fantasia is an open database created by the Massachusetts Institute of Technology in 1999 and was obtained on line. It contains the resting ECG recordings of 20 young (21–34 years old) and 20 elderly (68–85 years old) healthy subjects. We analysed data from 18 young subjects, to match the number of the patient dataset. ECG, respiratory rate and blood pressure recordings were made while the patients were lying supine and fairly still, and watching the Fantasia film.

### Data collection

A 12-lead ECG was used to collect heart rate data in the Fantasia group, and a LiDCOrapid^V2^ CNAP^®^ Module (CNSystems Medizintechnik AG, Graz, Austria) was used to record cardiovascular parameter data in the patient group. The latter consists of a finger sensor that measures arterial diameter by means of infrared light, and an inflatable cuff over the proximal phalanx of the middle and index fingers that monitors blood pressure. The CNAP system recorded heart rate and other cardiovascular parameters, with a sampling frequency of 100 Hz; in the control dataset the ECG was sampled at 150 Hz. Once the HR data was obtained, the HR time series of both patients and controls was resampled at 1 Hz for Poincaré plotting and analysis. Standardisation of the HR time series frequency was necessary to ensure homogeneity across datasets and to avoid artificially low HRV as a result of higher sampling frequencies (and vice versa). Standardisation is also necessary for Poincaré analysis, which relies on a uniform time difference between each time series point. In the patient group this was achieved by linear interpolation. In the control group this was achieved using the function ‘tach’ (WFDB toolbox for Matlab) [15, 16], which produces a uniformly sampled and smoothed instantaneous heart rate signal from heart-beat annotation files (the annotation files are available as part of the Fantasia dataset). Poincaré plots and SD1/2 measures were subsequently estimated from the HR data.

### Poincaré plot analysis

Data analysis was performed in MatLab r2016b [17] (MATLAB R2016b. Natick, Massachusetts: ^©^The MathWorks Inc., 2016).

Poincaré plots are non-linear and geometrical representations of HRV. The plot is a cluster of points along the line x = y (the line of identity), and every point on this line represents two heart beats of the exact equal rate, or successive identical RR intervals. Generally, any deviation above this line indicates an acceleration in heart rate form one beat to the next, and any deviation below it indicates a deceleration. The movement of points along the line represents long-term changes in heart rate. A wide, and long plot indicates high overall variability, which is indicative of a high level of autonomic tone, whereas narrow, bullet shaped plots indicate low HRV, and are typical of patients with a low level of autonomic function.

Poincaré plots can be analyzed both visually and geometrically. Visually, large, fan-shaped plots have been shown to indicate a prevalence of PSNS activity, whereas narrow and long, torpedo-like plots indicate a prevalence of SNS activity. Quantitatively, plots can be analyzed by fitting an ellipse to the distribution of points, and measuring the width of the distribution along (SD2) and perpendicularly to the identity line (SD1) [11, 12]. The ‘width’ of a Poincaré plot (SD1) has been described as a direct measure of beat-to-beat variability, and therefore parasympathetic activity [10, 13], with a wider SD1 indicating higher parasympathetic tone. Similarly, Brennan et al. [14] linked sympathovagal balance to the ‘length’ of the Poincaré plot (SD2) [18]. In this study, SD1 and SD2 are used to characterize HRV and, therefore autonomic function, in a young cohort of patients during surgery under propofol general anesthesia.

### Data extraction

HRV was quantified when patients were awake and during anesthesia using Poincaré plots and their respective SD1 and SD2 measures based on the subjects’ continuous heart rate readings. To this aim, two separate stages were extracted from each continuous recording of cardiovascular data:

1. Stage 1 (before anesthesia), which consists of 150–300 s window of HR data immediately preceding anesthetic induction;
2. Stage 2 (during anesthesia), which consists of a 300-s window of HR data taken between the 15th and 30th min of surgery.

Care was taken to extract time segments that did not involve the administration of fluid challenges, vasoactive or ionotropic drugs for Stage 2 in patients undergoing anesthesia. In the control group, two 300-s intervals were extracted at the same time points for comparison: 0 to 5 min, and 15–20 min. Values of heart rate and mean arterial pressure during Stage 1 and Stage 2 were also extracted for all patients.

HRV in each stage was analyzed using MatLab, using the Poincaré SD1/SD2 estimation functions described in Piskorski et al. [12]. Data was filtered to remove noise and ectopic beats, whereby data exceeding a threshold ± 20% were excluded, as recommended by Karlsson et al. [19]. The SD1 and SD2 parameters at Stage 1 and 2 were then compared. Significance was tested using a Mann–Whitney–Wilcoxon test (p < 0.05).

Furthermore, the real-time applicability of a Poincaré-based tool of HRV dynamics was investigated. This analysis was performed on post-hoc data for proof-of-concept. A ‘sliding window’ model was created in order to sequentially analyse data and therefore to build a model for a tool that can be applied on continuous, live data. The Poincaré plot parameters SD1 and SD2 were estimated over 20 s windows, which were updated every 5 s, therefore analysing data sequentially from start to end. Several possible window lengths for SD1/2 extraction were investigated, ranging from 5 to 120 s, to obtain the highest resolution possible while preserving accuracy. Through these investigations it was found that a 20 s window provided the highest resolution with lowest levels of noise. Windows of less than 20 s resulted in graphs with large amounts of noise, because the measurement of SD1 and SD2 was, in such cases, derived from 5 consecutive beats or less. On the other hand, windows longer than 20 s did not result in reduction of noise but displayed data with less time resolution; the shortest window possible was therefore selected to provide the highest resolution with minimal noise.

### Outcome measure

1. Estimation of SD1 (sympathetic tone—SNS) and SD2 (parasympathetic tone—PSNS) Poincaré parameters from HRV at baseline and during anesthesia.
2. Real-time modelling of HRV dynamics over the recording period, producing a model for autonomic monitoring in real-time.

## Results

### Poincaré plot quantification: SD1 and SD2

**Fig. 2:**
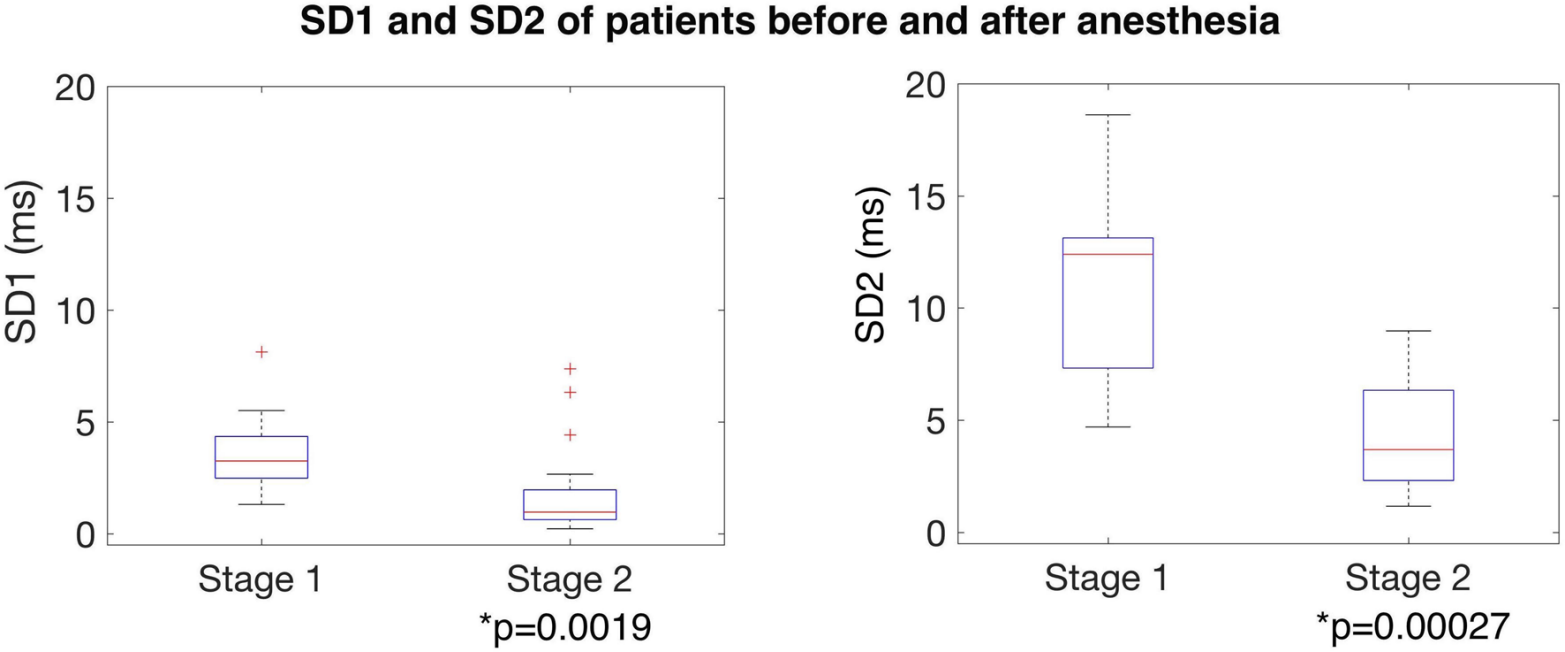
Boxplots of average SD1 and SD2 before (‘Stage 1’—left) and after (Stage 2’—right) anesthesia, indicating changes in parasympathetic and sympathetic tone between the two stages. *SD1* sympathetic function, *SD2* parasympathetic function

**Fig. 3:**
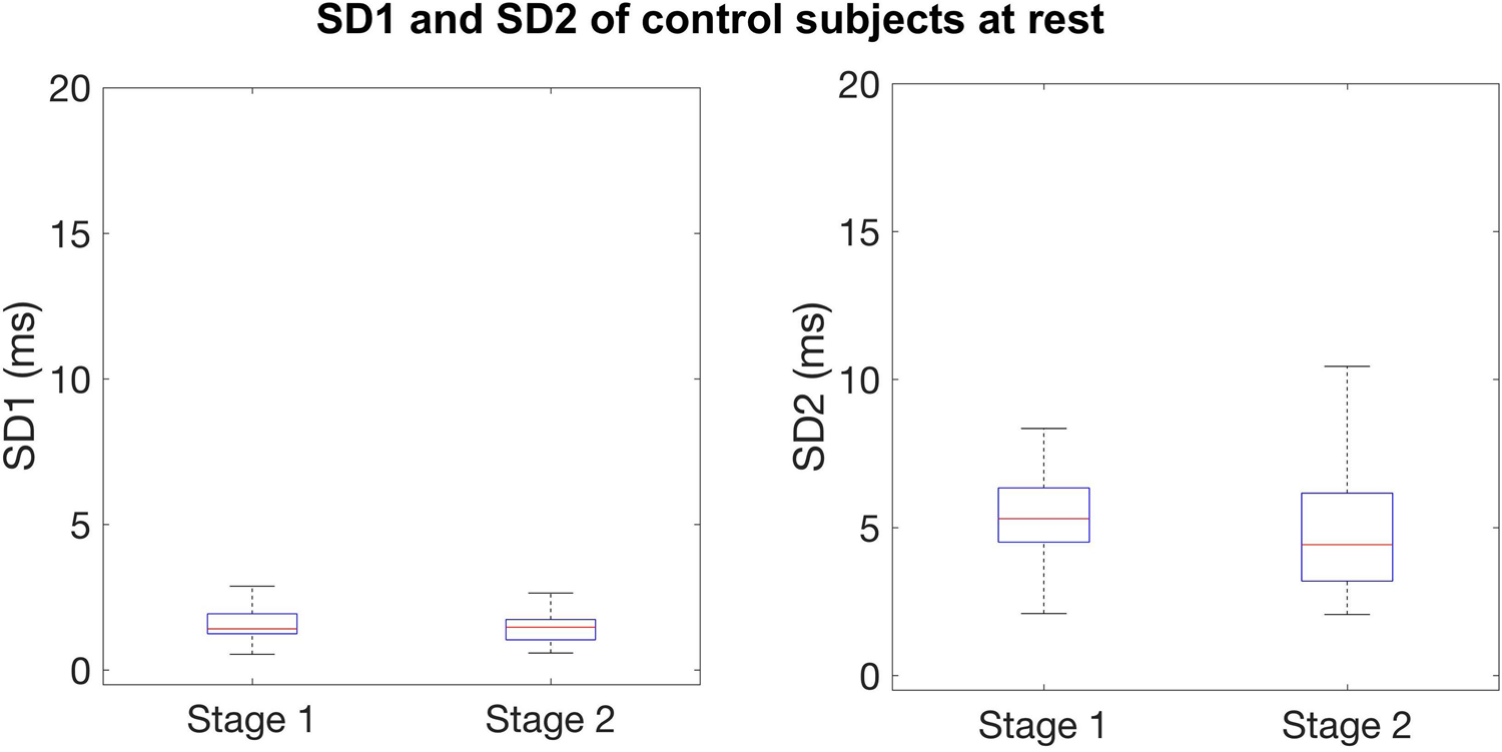
Boxplots of SD1 and SD2 from the resting controls taken at two time points during the recording, ‘Stage 1’ (left) and ‘Stage 2’ (right). *SD1* sympathetic function, *SD2* parasympathetic function

Quantification of Poincaré plots demonstrated visible changes in HRV (Fig. 1), which were mathematically quantifiable across the two stages. ‘Stage 1’ (baseline) SD1/2 were compared with ‘Stage 2’ values (during anesthesia). The comparison is shown in Fig. 2 and Table 1. Following anesthesia, both SD1 and SD2 values decreased, and these differences were significant (p = 0.019 and p = 0.00027), thus indicating significant suppression of both SNS and PSNS activity. HRV observed in resting controls was unchanged between Stage 1 and Stage 2 (Fig. 3) (Table 1).

**Table 1:** HRV quantification before (‘Stage 1’) and after (‘Stage 2’) anesthesia

	Mean	Median	IQR	p
Patient SD1				
Stage 1 (baseline)	3.53	3.24	1.86	0.019*
Stage 2 (maintenance)	1.86	0.96	1.33	
Patient SD2				
Stage 1 (baseline)	11.3	12.3	5.80	< 0.001*
Stage 2 (maintenance)	4.35	3.68	4.02	
Control SD1				
Stage 1 (0–5 min)	1.51	1.40	0.69	0.950
Stage 2 (15–20 min)	1.46	1.46	0.70	
Control SD2				
Stage 1 (0–5 min)	5.28	5.29	1.83	0.393
Stage 2 (15–20 min)	5.22	4.41	2.97	

Stage 1 anesthesia illustrates baseline data and it is defined as 5 min of recording prior to induction of anesthesia. Stage 2 anesthesia illustrates maintenance of anesthesia and it consists of 5 min taken during anesthesia. For the control data, Stage 1 consists of data from 0 to 5 min of the recording, and Stage 2 of data from 15 to 20 min of the recording. Significance was tested for using a Mann–Whitney–Wilcoxon *U*-test. Statistically significant difference are indicated with an asterisk

### Real-time monitoring: sliding window analysis

**Fig. 4:**
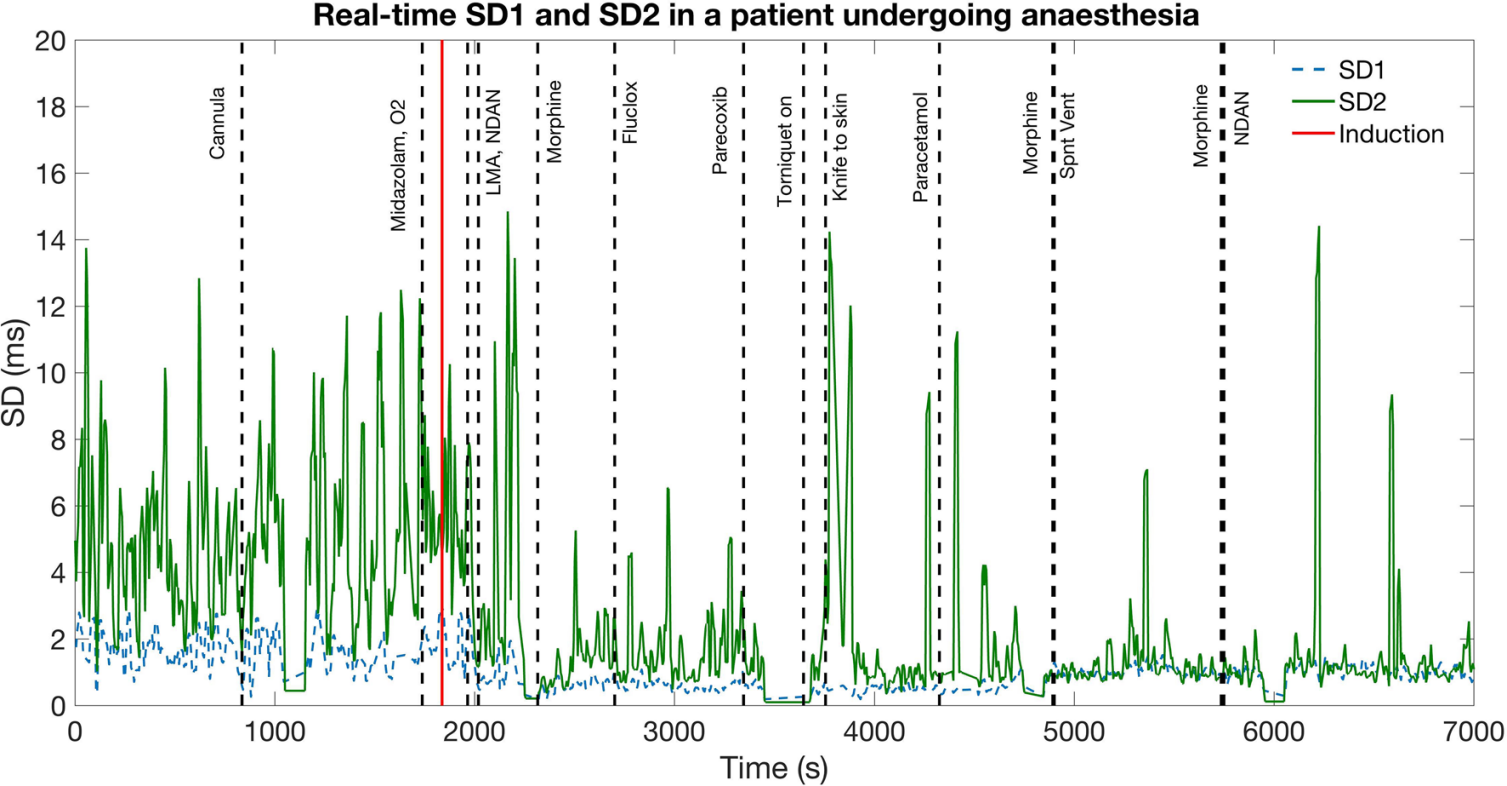
Sliding window analysis of SD1 and SD2 of a patient undergoing anesthesia using a 20-s window, indicating parasympathetic and sympathetic tone in real-time. *SD1* sympathetic function, *SD2* parasympathetic function. HRV is measured in SD, represenitng geometrical measures of distribution of the data points along the identity line. *LMA* laryingeal mark airway, *Fluclox* flucloxacillin, *NDAN* ondansentron

**Fig. 5:**
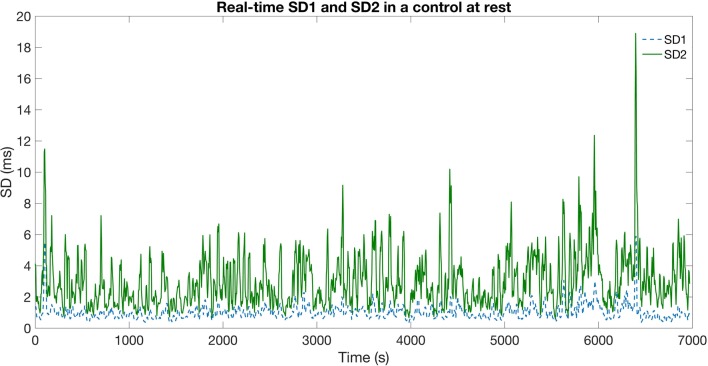
Sliding window analysis of SD1/2 of a control subject during a resting period using a 20-s window, indicating parasympathetic and sympathetic tone in real-time. *SD1* sympathetic function, *SD2* parasympathetic function. HRV is measured in SD, represenitng geometrical measures of distribution of the data points along the identity line

SD1 and SD2 were measured sequentially in 20-s windows sliding by 5-s, and plotted throughout the surgery for each patient. Examples of graphs using varying sampling length are provided in Supplementary Figs. 1–5. This was used to graphically model HRV and, therefore, autonomic function dynamically and in real-time. There was a marked decrease in, and lower variation of, both SD1 and SD2 following administration of propofol to patients (Fig. 4). Similar trends in SD1 and SD2 were not observed in the control group (Fig. 5).

## Discussion

**Fig. 6:**
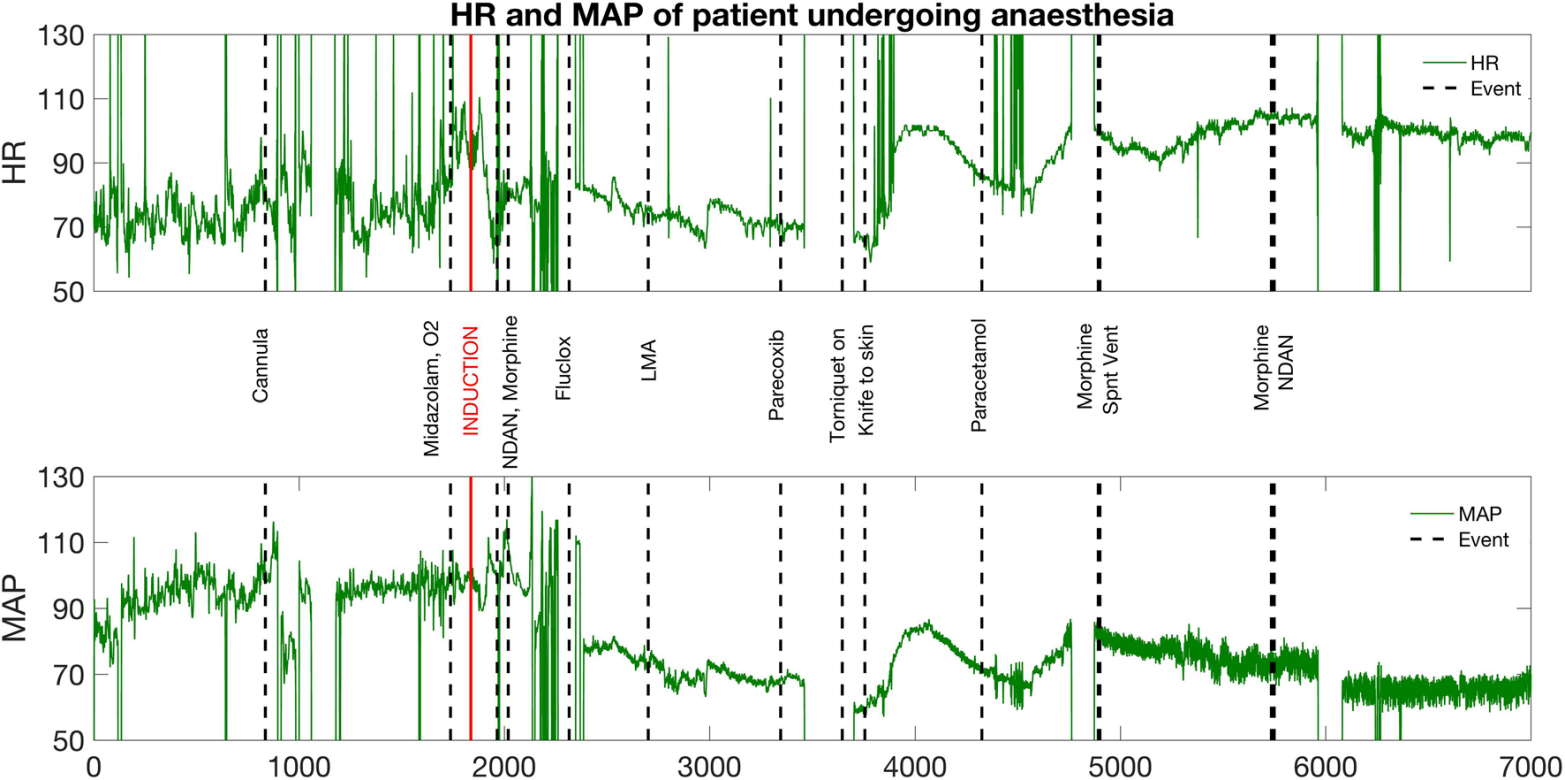
Continuous monitoring data for heart rate and mean arterial pressure corresponding to subject undergoing anaesthesia whose HRV monitoring data is depicted in Fig. 4. *LMA* laryingeal mark airway, *Fluclox* flucloxacillin, *NDAN* ondansentron

This study demonstrates a potential use of Poincaré analysis as a means to non-invasively characterize autonomic function in real-time. The shape of the Poincaré plots of the patients markedly converged following anesthesia in terms of ‘width’ and ‘length’, thus reflecting a decrease in both sympathetic and parasympathetic tone. This was quantified mathematically and could be visually appreciated on the real-time sliding window graph. Results from this study therefore validate the use of Poincaré plots for HRV quantification. This has been shown in the literature: Kamen et.al. [9] decisively validated Poincaré analysis of HRV trends on patients whose autonomic function was modulated pharmacologically or orthostatically: the width of the Poincaré plots was reduced during SNS-stimulating head-up tilt and anticholinergic atropine administration, but was increased after scopolamine, a known parasympathetic stimulant. Other than Poincaré plots, further methods of HRV analysis include time–frequency analysis, which is also commonly used. This involves power spectral density (PSD) analysis of electrocardiogram (ECG) waveform data, which categorizes HRV values into high frequency (HF), medium frequency (MF) and low frequency (LF) components with HF mainly reflecting PSNS activity, and LF the sympathovagal balance [20]. However, this is not a method that can be applied in real-time, and has been shown to be susceptible to high levels of respiratory noise [21]. Poincaré plots have been shown to be less affected by respiratory noise compared to other methods of heart rate variability analysis and correlate directly to PSD data [13, 14]: Poincaré width (SD2) reflects parasympathetic activation, and SD1 (length) reflects sympathetic antagonism to vagal tone. Furthermore, the SD1/SD2 ratio is analogous to the previously used spectral measure of LF/HF ratio, indicating sympathovagal balance. Hsu et al. [18] performed a retrospective study on patients undergoing anesthesia to assess Poincaré plots as a means of assessing ANS modulation, and compared these to time–frequency analysis. Similarly to Brennan et al., a correlation was found between Poincaré and spectral measures, but Poincaré was deemed more accurate and easily obtainable. Hsu et al. reported that the autonomic suppression observed using HRV of patients undergoing anesthesia was dynamic and reflective of the known autonomic depressant effect of propofol in real-time [22, 23], which is in line with reported findings of the present study. Despite the numerous validations of its significance, Poincaré analysis of HRV has not yet been used as a real-time tool for clinical purposes. Our study presents a first proof-of-concept and could, therefore, contribute to the development of a means to routinely and conveniently measure autonomic function in patients at the point of care. This provides a scope for personalization of anesthetic protocols and pre-surgical risk stratification. This is especially relevant in light of the increasing number of studies that have begun to investigate and prove the value of HRV in predicting complications of anesthesia and surgery [24, 25] (Fig. 6).

There are several limitations to consider. Firstly, the real-time analysis model was applied to data following, and not during, data collection. The designed tool was able to reflect changes in autonomic function in real-time graph by analysing data on a window-by-window basis. It therefore analysed the data sequentially as if in real-time, but the data used for the analysis had been pre-collected. Following positive results from this retrospective proof-of-concept data, further adjustment of the system will be needed to apply the tool to truly real-time data at the point of collection, rather than retrospectively. This study therefore provides a model, and not a finished product, for the analysis of data in real-time. However, it is important to note that the proposed HRV analysis model is transferable to live data without the need for developing a specialized module, via a direct interface between the patient monitor and a laptop device, where the HR data can be streamed and analysed directly, and an SD1/2-based index displayed on the laptop monitor. Software for such direct monitor-laptop interface already exist, e.g. VSCapture (open source software, https://sourceforge.net/projects/vscapture/). Additional software may need to be implemented in order to calculate the SD1/2 indices, e.g., in Python (Python Software Foundation, https://www.python.org/). After an initial delay of 20-s (to buffer sufficient data as per the analysis presented in our work) the index can be updated every second as per the model presented in this study. Secondly, the analysis tool that was constructed is based on continuous analysis of Poincaré plots derived from HR data. The plots constructed, therefore, reflect the relationship between two consecutive HR values. The HRV analysis was therefore based on pulse rate variability (PRV); this has been validated as a feasible and equivalent alternative to HRV calculation by RR-interval analysis for the analysis of HR data derived form pletysmography [26–28]. The rationale behind using HR estimated by the LidCO CNAP monitoring system and not using ECG-derived R–R intervals is mainly for reducing algorithmic complexity and improving accuracy. Estimation of R–R intervals from ECG requires additional analysis to estimate the exact locations of the QRS complexes and identification of the fiducial points, which is non-trivial in noisy or low amplitude ECGs. This is particularly important considering that recordings are performed in the noisy environment of the operating theatre where ECG sensors are susceptible to artifacts such as electrode movement (which introduces baseline wander in the measurements) and noise from the use of various surgical equipment. The HR data derived from the LiDCO CNAP finger sensor is less noisy and using this data directly allows a more flexible, cheaper and less complex ‘plug-and-play’ implementation. However, a limitation is that the SD1 and SD2 variables obtained from HR and RR intervals are not directly comparable. The conversion of HR data to RR interval data is relatively simple, but this would have added a further layer of complexity and, subsequently, potential error as the data would still reflect HR-derived RR intervals and not true RR intervals. It remains important to consider that differences in recording methods (12-lead ECG versus LidCO CNAP by finger plethysmography) may have given rise to intrinsic variation in the heart rate readings between controls and subjects. A further potential limitation of the study rests in the sampling frequencies of the data collection methods. The CNAP system records cardiovascular parameters with a sampling rate of 100 Hz. Even though sampling frequencies greater than 250 Hz are generally recommended for HRV analysis (from ECG R–R intervals), Mahdiani and colleagues have shown that a sampling rate as low as 50 Hz could be used for measuring the ECG signal without compromising the accuracy of the calculated time domain HRV parameters [29]. Nevertheless, increasing the sampling frequency of the input data is a factor that warrants further investigation. Furthermore, before its clinical application, comparison to other previously validated tools available for assessment the ANS (e.g., state entropy, response entropy, Surgical Pleth Index [30] or Analgesia Nociception Index [31]), subtraction of the respiratory effect on HRV and further refining of the noise-reduction filter may also be warranted.

Consideration must be given to how patient morbidity may affect HRV. For example, conditions such as heart failure [32] and use of some medications [33] are likely to change HRV dynamics, and the impact of such variables investigated. During anesthesia itself, several factors may also affect HRV. Cardioactive agents (atropine, ephedrine), fluid boluses, and the effect of operative events on HRV must be considered, as these agents exert their effects by direct modulation of the autonomic nervous system, and are therefore likely to have a profound effect on HRV. The operating theatre is an inherently noisy environment, with artefacts arising from the procedure itself, such as intubation, knife to skin, diathermy etc., that are difficult and sometimes impossible (particularly in the case of diathermy) to remove. This is an issue even with commercially available systems, e.g., the BIS monitor stops displaying an index when diathermy is used. However, any measures derived from heart rate variability, such as SD1 and SD2, reflect the continuous modulation of the sino-atrial node and are, thus, bound to display some additional physiologically-related variation.

The ability to non-invasively and continuously measure HRV in real-time can be useful in clinical practice. The proposed tool has the potential to be employed in a continuous real-time monitoring system during anesthesia within the operating theatre, providing the anesthetist with direct, quantitative information about autonomic tone in real-time. Future development of this tool involves its trial on live data during collection, and the evaluation of its value as a pre- and in-surgical assessor and predictor of complications [34].

Our findings support the feasibility of Poincaré plot analysis for use in the development of a dynamic, non-invasive and real-time autonomic function characterization during anesthesia via the HRV. Such a tool would provide a faster, easier and more accessible ANS monitoring tool than the currently available methods of testing and, pending further development, has a wide scope for potential clinical applications.

## Electronic supplementary material

Below is the link to the electronic supplementary material.

**Supplementary Fig. S1** Sliding window analysis of SD1 and SD2 of a patient undergoing anesthesia using a 5-second window, indicating parasympathetic and sympathetic tone in real-time. SD1 = sympathetic function and SD2 = parasympathetic function. Supplementary material 1 (JPG 598 KB)

**Supplementary Fig. S2** Sliding window analysis of SD1 and SD2 of a patient undergoing anesthesia using a 20-second window, indicating parasympathetic and sympathetic tone in real-time. SD1 = sympathetic function and SD2 = parasympathetic function. Supplementary material 2 (JPG 657 KB)

**Supplementary Fig. S3** Sliding window analysis of SD1 and SD2 of a patient undergoing anesthesia using a 40-second window, indicating parasympathetic and sympathetic tone in real-time. SD1 = sympathetic function and SD2 = parasympathetic function. Supplementary material 3 (JPG 700 KB)

**Supplementary Fig. S4** Sliding window analysis of SD1 and SD2 of a patient undergoing anesthesia using a 60-second window, indicating parasympathetic and sympathetic tone in real-time. SD1 = sympathetic function and SD2 = parasympathetic function. Supplementary material 4 (JPG 585 KB)

**Supplementary Fig. S5** Sliding window analysis of SD1 and SD2 of a patient undergoing anesthesia using a 120-second window, indicating parasympathetic and sympathetic tone in real-time. SD1 = sympathetic function and SD2 = parasympathetic function. Supplementary material 5 (JPG 524 KB)
