# Supplementary figures and images for: Non-invasive real-time autonomic function characterization during surgery via continuous Poincaré quantification of heart rate variability
Source: J Clin Monit Comput. 2018 Oct 3;33(4):627–35. doi: 10.1007/s10877-018-0206-4 (PMC6602980; doi:10.1007/s10877-018-0206-4)

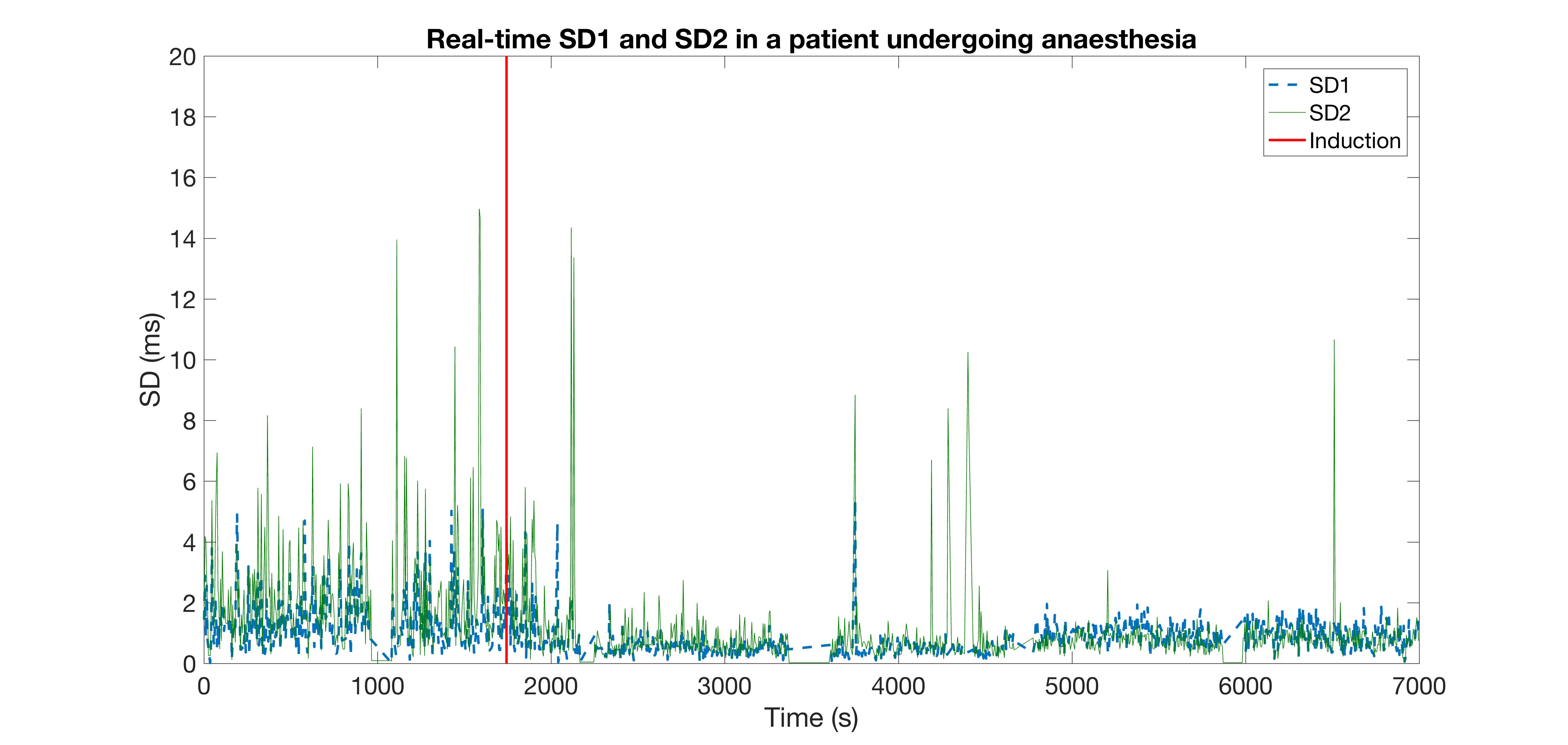

Supplement: Supplementary file 1 — Supplementary Fig. S1 Sliding window analysis of SD1 and SD2 of a patient undergoing anesthesia using a 5-second window, indicating parasympathetic and sympathetic tone in real-time. SD1 = sympathetic function and SD2 = parasympathetic function. Supplementary material 1 (JPG 598 KB) [file 10877_2018_206_MOESM1_ESM.jpg]

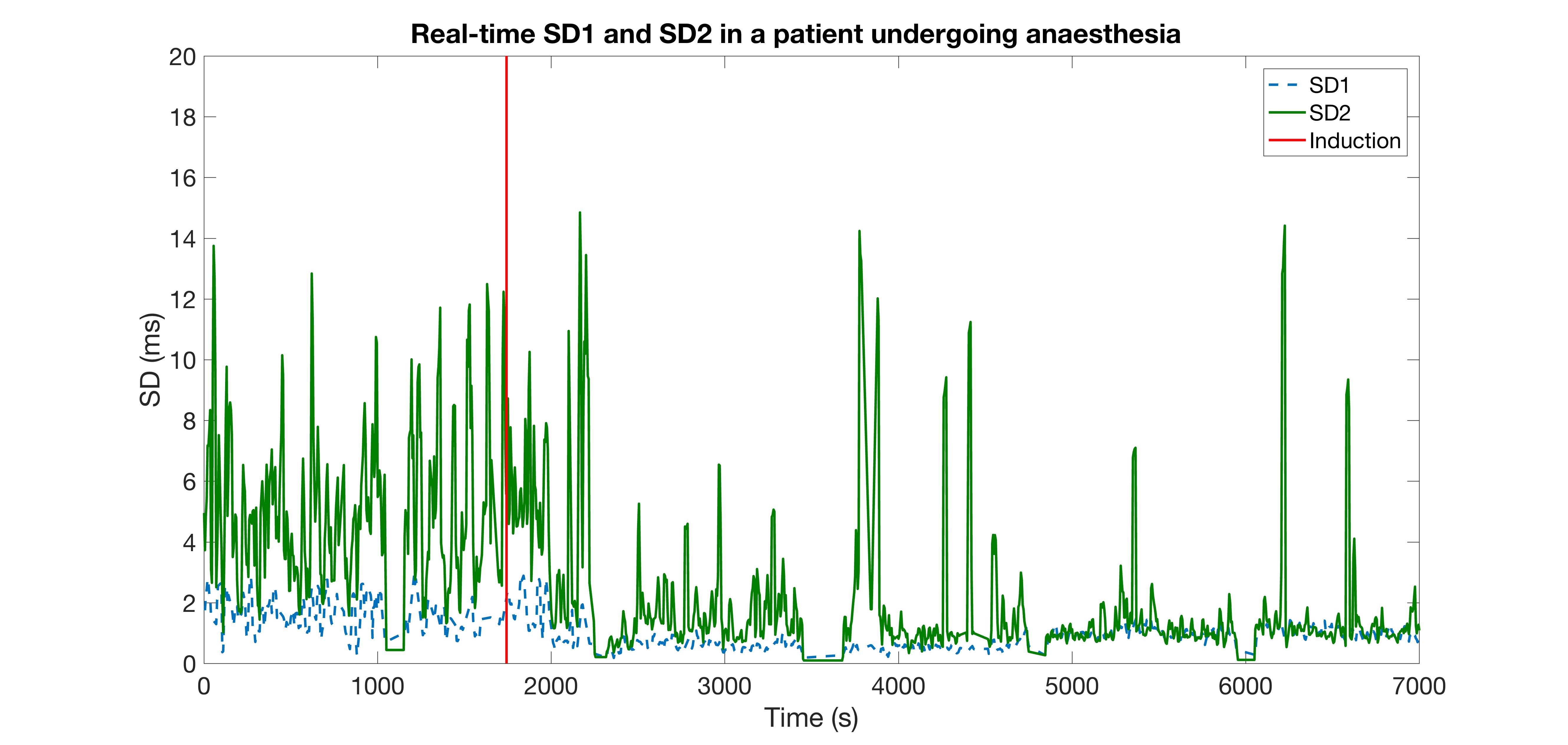

Supplement: Supplementary file 2 — Supplementary Fig. S2 Sliding window analysis of SD1 and SD2 of a patient undergoing anesthesia using a 20-second window, indicating parasympathetic and sympathetic tone in real-time. SD1 = sympathetic function and SD2 = parasympathetic function. Supplementary material 2 (JPG 657 KB) [file 10877_2018_206_MOESM2_ESM.jpg]

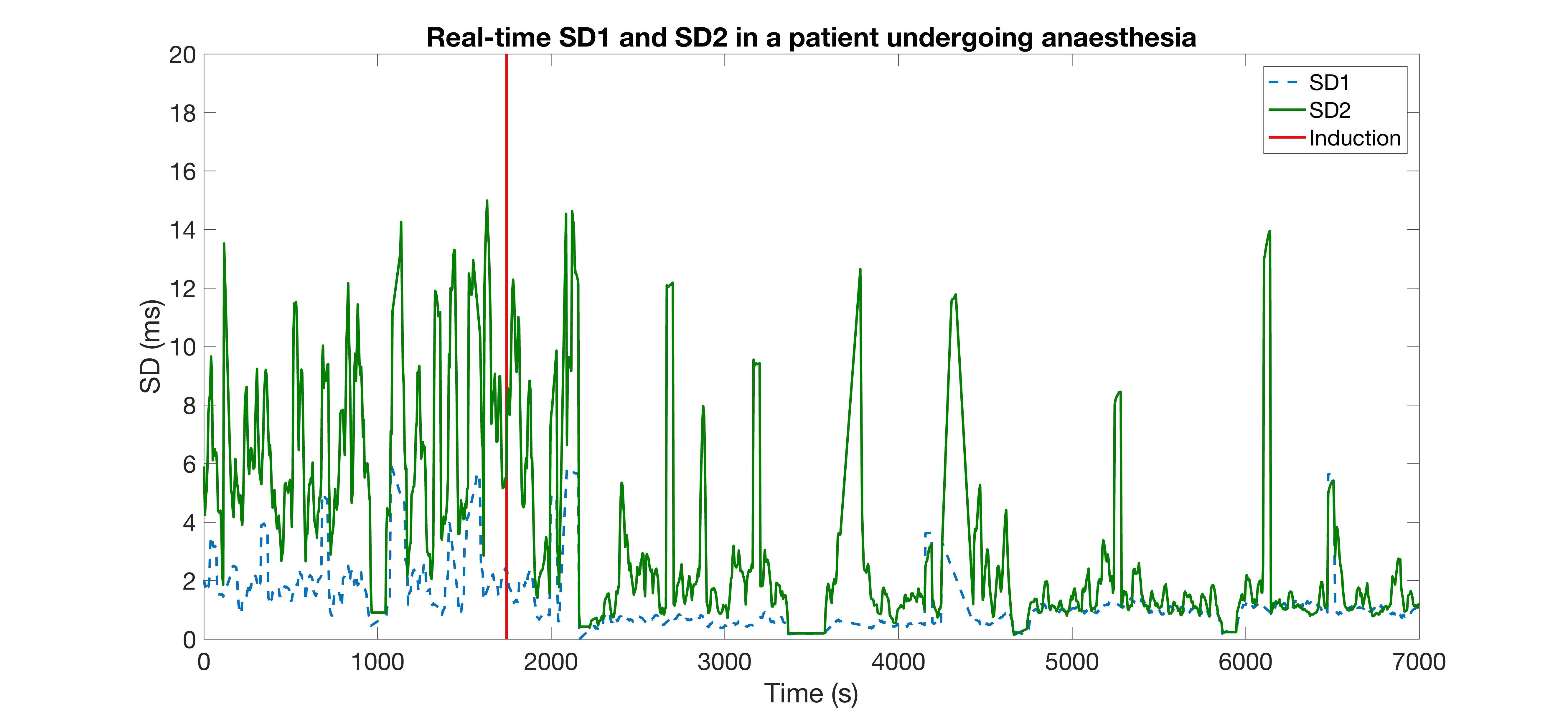

Supplement: Supplementary file 3 — Supplementary Fig. S3 Sliding window analysis of SD1 and SD2 of a patient undergoing anesthesia using a 40-second window, indicating parasympathetic and sympathetic tone in real-time. SD1 = sympathetic function and SD2 = parasympathetic function. Supplementary material 3 (JPG 700 KB) [file 10877_2018_206_MOESM3_ESM.jpg]

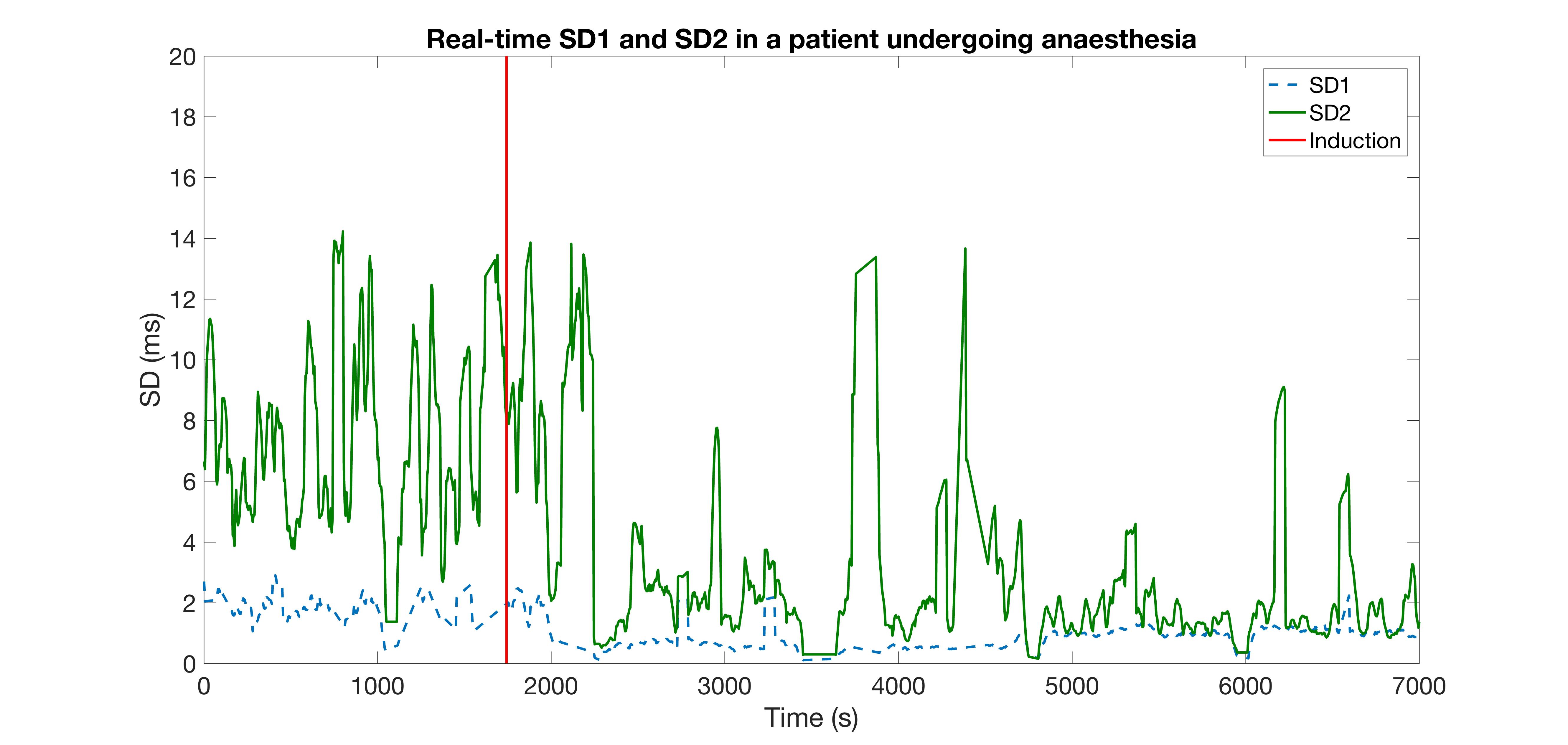

Supplement: Supplementary file 4 — Supplementary Fig. S4 Sliding window analysis of SD1 and SD2 of a patient undergoing anesthesia using a 60-second window, indicating parasympathetic and sympathetic tone in real-time. SD1 = sympathetic function and SD2 = parasympathetic function. Supplementary material 4 (JPG 585 KB) [file 10877_2018_206_MOESM4_ESM.jpg]

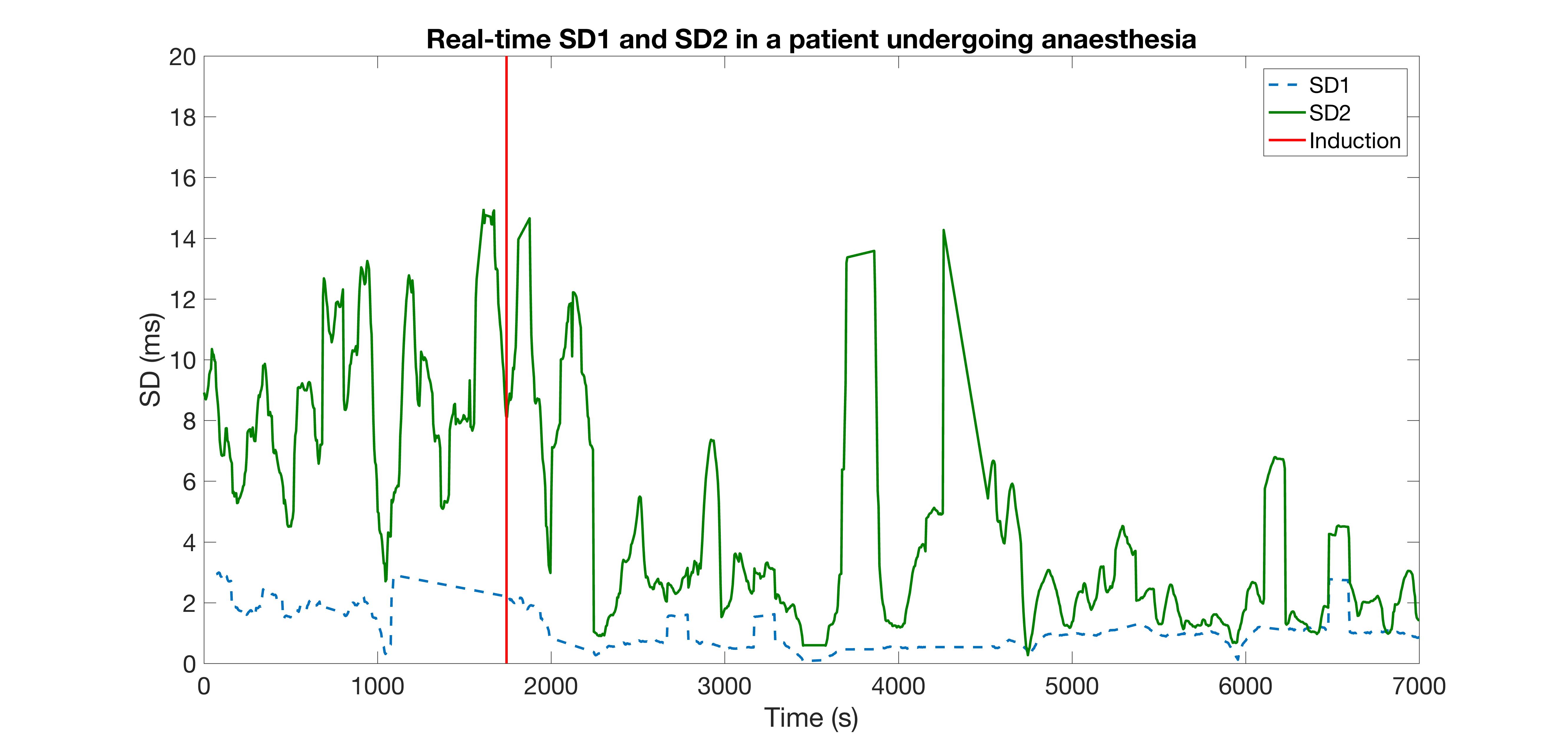

Supplement: Supplementary file 5 — Supplementary Fig. S5 Sliding window analysis of SD1 and SD2 of a patient undergoing anesthesia using a 120-second window, indicating parasympathetic and sympathetic tone in real-time. SD1 = sympathetic function and SD2 = parasympathetic function. Supplementary material 5 (JPG 524 KB) [file 10877_2018_206_MOESM5_ESM.jpg]
